# Management des konvulsiven Status epilepticus im Kindesalter

**DOI:** 10.1007/s00508-025-02570-2

**Published:** 2025-09-16

**Authors:** Sarah Glatter, Francesco Cardona, Eugen Trinka, Markus Leitinger, Martha Feucht

**Affiliations:** 1 Department of Pediatrics and Adolescent Medicine, Center for Rare and Complex Childhood Onset Epilepsies, Member of ERN EpiCARE, Medical University Vienna, Vienna, Austria; 2 Department of Pediatrics and Adolescent Medicine, Division of Neonatology, Pediatric Intensive Care and Neuropediatrics, Medical University Vienna, Vienna, Austria; 3 Department of Neurology, Christian Doppler University Hospital, Member of ERN EpiCARE, Paracelsus Medical University, Salzburg, Austria; 4 Neuroscience Institute, Christian Doppler University Hospital, Centre for Cognitive Neuroscience, Paracelsus Medical University, Salzburg, Austria; 5 Karl Landsteiner Institute, Clinical Neurosciences, Salzburg, Austria

**Keywords:** Pädiatrischer Status epilepticus, Pädiatrische Neurologie, Anfallsmanagement, Konvulsive Anfälle, Anfallssupprimierende Medikamente (ASMs), Pediatric status epilepticus, Pediatric neurology, Seizure management, Convulsive seizures, Antiseizure medications (ASMs)

## Abstract

Konvulsive Status epileptici (KSE) gehören im Kindesalter zu den häufigsten Notfällen. KSE sind zeitkritisch, wobei mit zunehmender Dauer die Beendigung des KSE schwieriger bzw. unwahrscheinlicher wird und sowohl Mortalität als auch unmittelbare und Langzeitkomplikationen exponentiell zunehmen. Zügige Diagnostik und Behandlung pädiatrischer KSE (PKSE) sind daher essenziell, aktualisierte allgemein akzeptierte Leitlinien für strukturiertes Vorgehen existieren – im Gegensatz zum Erwachsenenalter – jedoch derzeit nicht. Am EpiCare-Zentrum der Universitätsklinik für Kinder- und Jugendheilkunde Wien wurde, basierend auf aktueller Evidenz, in enger Kooperation mit der Universitätsklinik für Neurologie Salzburg ein Konzept erarbeitet, das eine kontinuierliche Verlaufsdokumentation – beginnend mit dem Zeitpunkt, zu dem der junge Patient zuletzt symptomfrei gesehen wurde über prähospitale Interventionen bis hin zum Eintreffen in der Notfallambulanz und weiters bis zur Aufnahme an der pädiatrischen Intermediate Care(IMC)- oder Intensiv-Station (PICU) – ermöglicht. Des Weiteren wurde eine „Pocket Card“ mit detaillierten Informationen über strukturierte spezifische Diagnostik und Behandlung erstellt, die unabhängig von der ärztlichen Besetzung (z. B. im Nachtdienst und an Wochenenden) rasche adäquate Maßnahmen garantieren soll. Das hier vorgestellte Konzept soll zudem Rettungskräfte und NotärztInnen sowie Erstaufnahmezentren im Umfeld sensibilisieren sowie Abläufe harmonisieren und so dazu beitragen, Übergänge in (super)refraktäre KSE mit u. U. irreversiblen Konsequenzen hintanzuhalten.

## Abstract (en)

Convulsive status epilepticus (CSE) is one of the most common pediatric emergencies. The duration of CSE is critical, whereby with increasing duration the cessation of CSE becomes more difficult or less probable and mortality as well as short and long-term complications increase exponentially. Prompt diagnostics and treatment of pediatric CSE (PCSE) are therefore essential. In contrast to adulthood, updated generally accepted guidelines for a structured approach do not yet exist. Based on current evidence the Austrian EpiCARE centers of the Department of Pediatrics at the Medical University Vienna in close cooperation with the Department of Neurology at the Christian Doppler University Hospital in Salzburg developed a continuous documentation process beginning with the time when the young patient was last seen free of symptoms, through prehospital interventions up to admission in the emergency outpatient department and further up to the admission in the pediatric intermediate care (IMC) or intensive care units (PIC). In addition, a pocket card with detailed information on structured specific diagnostics and treatment was developed, which is aimed at guaranteeing rapid adequate measures independent of the staffing by medical personnel (e.g., during night shifts or at weekends). The concept presented here is aimed at sensitizing prehospital and emergency physicians as well as pediatricians at local initial admission centers to further harmonize in-/inter-house treatment standards with the intention of preventing refractory or superrefractory PCSE and possible resulting complications.

Pädiatrische konvulsive Status epileptici (PKSE) gehören zu den häufigsten Notfällen im Kindesalter [1]. PKSE sind mit signifikant erhöhtem Mortalitäts- und Morbiditätsrisiko assoziiert, wobei mit zunehmender Dauer die Beendigung immer schwieriger bzw. unwahrscheinlicher wird und die Wahrscheinlichkeit sowohl für unmittelbare als auch für Langzeitkomplikationen exponentiell zunimmt [2–10].

Die Dauer des PKSE ist zudem – neben der richtigen Dosierung anfallssupprimierender Medikamente (ASMs) – die einzige durch die Medizin beeinflussbare Outcome-Variable [7, 9–12].

Zügige (Differenzial‑)Diagnostik und frühe Behandlung sind somit essenziell, um irreversible strukturelle Hirnschäden, permanente funktionelle Defizite und die Änderung neuronaler Netzwerke mit nachfolgend schwer behandelbaren Epilepsien zu minimieren [13, 14].

Der Wettlauf mit der Zeit stellt jedoch – insbesondere dann, wenn der PKSE de novo und außerhalb des Spitals beginnt – eine Herausforderung dar. Logistische und strukturelle Hindernisse führen hier jedoch oft zu beträchtlichen Verzögerungen, bzw. ist ein signifikanter Prozentsatz der betroffenen Kinder bereits bei Eintreffen in der Notfallambulanz des nächstgelegenen Spitals definitionsgemäß als refraktär zu bezeichnen [4, 9, 12, 15–18].

Kontrollierte prospektive Studien pädiatrischer PatientInnen sind rar, und aktualisierte allgemein akzeptierte Leitlinien oder zumindest Empfehlungen für strukturierte Diagnostik und Therapie existieren – im Gegensatz zum Erwachsenenalter – nicht. Dies führt lokal zu höchst unterschiedlichem und teilweise nur bedingt effizientem Vorgehen [4, 10, 12, 14, 15, 19].

Am pädiatrischen EpiCARE-Zentrum der Universitätsklinik für Kinder- und Jugendheilkunde Wien wurde daher nach Durchsicht und Bewertung der ab dem Jahr 2000 zu PKSE publizierten Literatur in MEDLINE durch 2 Kinder- und einen pädiatrischen Intensivmediziner, basierend auf aktueller Evidenz und in enger Kooperation mit dem EpiCARE-Zentrum der Universitätsklinik für Neurologie Salzburg, ein Konzept erarbeitet, das eine kontinuierliche Verlaufsdokumentation – beginnend mit dem Zeitpunkt, zu dem der junge Patient zuletzt symptomfrei gesehen wurde über prähospital erfolgte Interventionen bis hin zum Eintreffen in der Notfallambulanz und weiters bis zur Aufnahme an der pädiatrischen Intermediate Care(IMC)- oder Intensiv-Station (PICU) – ermöglicht.

Des Weiteren wurde eine „Pocket Card“ mit detaillierten Informationen über strukturierte spezifische Behandlung und Diagnostik im Spital erstellt, die unabhängig von der ärztlichen Besetzung auch außerhalb der Kernarbeitszeit (z. B. auch im Nachtdienst und an Wochenenden) rasche und adäquate Maßnahmen garantieren soll.

Das hier vorgestellte Konzept soll dazu führen, sowohl Diagnostik- und Behandlungs-Abläufe an der eigenen Klinik zu optimieren als auch – in Zusammenarbeit mit Rettungsdiensten und umliegenden Kinderabteilungen (insbesondere jenen mit geringeren strukturellen Ressourcen) – das Management von PKSE zu harmonisieren, um Übergänge in (super)refraktäre PKSE zu minimieren [3, 9, 12, 20].

Nichtkonvulsive und neonatale pädiatrische SE sind nicht Gegenstand dieser Publikation, da sie aufgrund differenter diagnostischer und therapeutischer Voraussetzungen andere Vorgaben verlangen [5, 15, 21].

## Pathophysiologie, Definition und Klassifikation

### Pathophysiologie

Veränderungen der Expression von γ‑Aminobuttersäure A (GABA-A), N‑Methyl-D-Aspartat (NMDA) und α‑Amino-3-hydroxy-5-methylisoxazol-4-propionsäure(AMPA)-Rezeptoren sowie Störungen intra- und extrazellulärer Ionenverteilung führen zum Versagen anfallsterminierender und/oder zur Initiierung anfallsverlängernder Mechanismen und dadurch zum Auftreten einzelner prolongierter epileptischer Anfallsaktivität oder zu Anfallsserien [13, 22, 23].

### Definition (Abb. 1)

Die Internationale Liga gegen Epilepsie (ILAE) führte 2015 eine mittlerweile allgemein akzeptierte Definition und Klassifikation des Status epilepticus (SE) ein, die ein klares Zeitkonzept bezüglich Diagnosestellung und Therapiebeginn (Zeitpunkt T1), aber auch die Zeitspanne für die erfolgreiche Behandlung zur Vermeidung konsekutiver Schäden (Zeitpunkt T2) – unter Berücksichtigung verschiedener Statustypen – vorschlägt [13, 24]. Damit wird dem Umstand Rechnung getragen, dass ab einem bestimmten Zeitpunkt die Wahrscheinlichkeit spontaner Remission exponentiell signifikant abnimmt, das Ansprechen auf therapeutische Maßnahmen immer geringer wird und somit das Mortalitäts- bzw. Morbiditätsrisiko zunimmt [4, 13, 15, 17, 22].

**Abb. 1:**
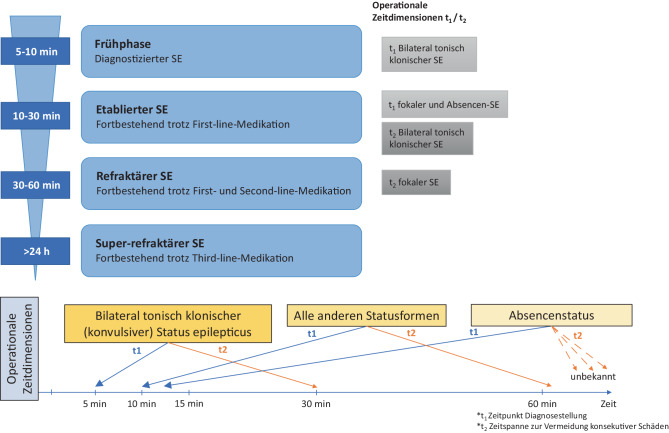
Definition des Status epilepticus (SE)

*T1* bezeichnet den Zeitpunkt der Überschreitung der üblichen Dauer eines epileptischen Anfalls und somit der Notwendigkeit der Initiierung gezielter Therapiemaßnahmen:

- *früher („diagnostizierter“) SE*: Dauer > 5 min für bilateral tonisch klonische Anfälle; > 10 min für fokale Anfälle mit/ohne Bewusstseinsstörung sowie 10–15 min für Absencen,
- *etablierter („Benzodiazepin-refraktärer“) SE*: Dauer > 5 min + kein Effekt der Erstlinienmedikation.

*T2* bezeichnet die Zeitspanne gerechnet ab Beginn des SE, innerhalb der im Idealfall, um Folgeschäden zu vermeiden, gezielte Behandlung spätestens erfolgreich sein sollte: 30 min für bilateral tonisch klonische Anfälle, > 60 min für fokale SE (FSE). Für Absencen-SE ist die vorliegende Evidenz unzureichend.

- *Refraktärer SE (RSE)*: Persistieren der Anfallsaktivität (klinisch und/oder im EEG) trotz Gabe von Erst- und Zweitlinienmedikation. Zur Behandlung des RSE sind Anästhetika als Drittlinienmedikation notwendig.
  - *„New-onset refractory status epilepticus“ (NORSE)* ist definiert als RSE, der als Erstmanifestation und ohne vorbestehende Epilepsie und/oder neurologische Erkrankung auftritt [25–30].
    - *„Febrile infection-related epilepsy syndrome“ (FIRES)* ist eine Unterkategorie von NORSE, bei der ein fieberhafter Infekt 2 Wochen bis 24 h vor dem SE auftritt.
- *Superrefraktärer SE (SRSE)* liegt vor, wenn Anfallsaktivität ≥ 24 h trotz Allgemeinanästhesie („Komatherapie“) andauert oder rezidiviert, wenn diese zurückgenommen/beendet wird [13, 15, 24, 31, 32].

### Klassifikation (Abb. 2)

Die Klassifikation des SE erfolgt in 4 Achsen: Semiologie (Anfallstypen), Ätiologie, elektroenzephalographische (EEG) Korrelate und Alter [13, 22, 24].

- Hauptkriterien der Unterteilung von Achse 1 sind in erster Linie das Vorhandensein prominenter motorischer Phänomene und zur weiteren Unterteilung die Bewusstseinslage sowie die Zuordnung entsprechend der ILAE-Anfallsklassifikation [33].
- Die Einteilung von Achse 2 unterscheidet KSE mit bekannter (akuter, zurückliegender, progredienter) Ätiologie, KSE im Rahmen von elektroklinischen Syndromen und KSE unbekannter/kryptogener Ursache [13, 24, 34].
- Achse 3 beschreibt assoziierte EEG-Veränderungen. Im Gegensatz zum Erwachsenenalter ist die Datenlage für PKSE – insbesondere auch im Hinblick auf die prognostische Wertigkeit des EEG – noch unzureichend [35–39].
- Achse 4 umfasst folgende Altersgruppen: Neugeborenen-Periode (bis 30. Lebenstag), Kleinkindalter (1 Monat bis 2 Jahre), Kindheit (über 2 Jahre bis 12 Jahre), Adoleszenz (über 12 bis 19 Jahre), Erwachsene und höheres Lebensalter (60 Jahre oder mehr). Aktuelle internationale Leitlinien für (Klein‑)Kinder existieren derzeit nicht [15].
  - Rezent aktualisierte Empfehlungen existieren derzeit ausschließlich für das Neugeborenenalter [21].

**Abb. 2:**
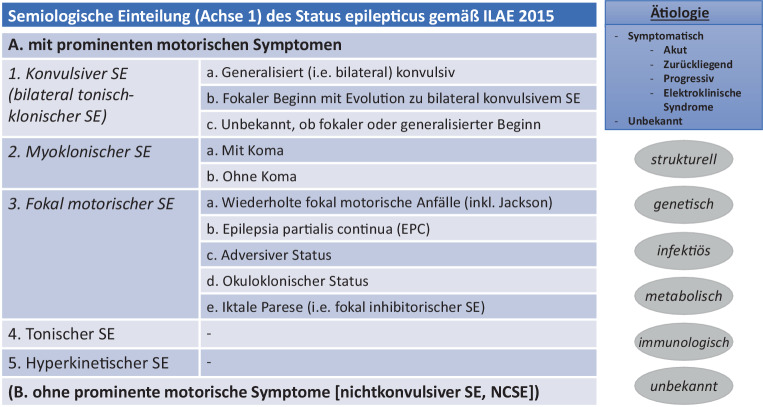
Klassifikation des Status epilepticus (SE)

## Epidemiologie

Abhängig von verfügbaren Ressourcen in unterschiedlichen Ländern und Regionen variieren die publizierten Jahresinzidenzraten für PKSE von 3 bis 58/100.000. Die Inzidenz ist geschlechtsunabhängig [5, 6, 18, 40–43].

PKSE beginnen in jeweils 50 % entweder fokal oder sind von Beginn an generalisiert, während nur ein geringer Prozentsatz der SE (11–29 %) durchgehend fokal bleibt [40].

Etwa 25 % aller Kinder mit Epilepsie haben im Verlauf einen oder mehrere PKSE, 10 % dieser Kinder erstmanifestieren als PKSE, in 60 % sind diese Kinder vorher gesund. Umgekehrt haben 27–35 % der Kinder mit Epilepsie im Verlauf einen oder wiederholte PKSE [5, 42].

Das Gesamtrezidivrisiko nach erstem PKSE beträgt 20 % nach 4 Jahren, ist jedoch individuell abhängig von den zugrunde liegenden Ursachen (3 % für febrile SE, 4 % bei unbekannter, 11 % bei akut symptomatischer, 44 % bei entfernt symptomatischer und 67 % bei progressiv symptomatischer Ätiologie). Etwa 69 % der Rezidive treten innerhalb eines Jahres nach der ersten Episode auf [5, 41, 42].

In der Mehrzahl der PKSE ist die Ätiologie symptomatisch, in 7–10 % der Fälle bleiben die Ursachen trotz modernster diagnostischer Hilfsmittel weiterhin unklar [6].

Die überwiegende Mehrzahl der PKSE tritt – aufgrund höherer Suszeptibilität des Gehirns für (v. a. Fieber-assoziierte) Anfälle – vor dem 2. Lebensjahr mit einer Inzidenz von 51–156/100.000/Jahr in dieser Altersgruppe auf. In dieser Altersgruppe ist, bedingt durch prognostisch ungünstige Ursachen (metabolisch und/oder genetisch), auch der Anteil an RSE am höchsten. Neben Alter und Ätiologie sind bestimmte Syndrome (z. B. Dravet-Syndrom, Lennox-Gastaut-Syndrom, FIRES etc.) mit RSE assoziiert [2, 6, 40].

Die Inzidenz für RSE beträgt 12–40 %, jene für SRSE 10–15 % aller PKSE [40].

Das Risiko für eine dem PKSE nachfolgende chronische Epilepsie innerhalb von 2 Jahren beträgt 25–40 % und entspricht damit dem Rezidivrisiko nach einem ersten unprovozierten Anfall [4–6, 21–23, 25, 28, 42–44].

## Diagnostik und Management des PKSE

Wie bei Erwachsenen ist die Dauer vom frühestmöglichen Beginn bis zur erfolgreichen Beendigung des PKSE die wichtigste medizinische beeinflussbare Outcome-Variable. Dies erfordert ein strukturiertes Konzept, in dem Diagnostik und Behandlung parallel und zügig erfolgen [4, 7, 10, 12, 15].

In einem ersten Schritt sind Vitalparameter/klinische Stabilisierung (ABCDE-Schema) [45] und Schutz vor Selbstgefährdung zu gewährleisten. Danach müssen – primär klinisch – die Abgrenzung gegenüber nichtepileptischen Phänomenen, z. B. funktionell-dissoziativem Status, sowie die Klassifikation des PKSE erfolgen. Der zeitnahe Zugang zu konventionellem EEG innerhalb 1 h (24 h/7 Tage pro Woche) und kontinuierlichem EEG-Monitoring für mindestens 24–48 h an der IMC/PICU zur Unterstützung von Definition/Klassifikation, aber auch zur Überwachung der nachhaltigen Wirksamkeit therapeutischer Maßnahmen (z. B. bei dem PKSE möglich nachfolgenden non-konvulsiven Anfällen) werden zunehmend empfohlen, bleiben derzeit jedoch fast ausschließlich Grad-IV- und -V-Zentren vorbehalten [6, 16, 36, 37, 39, 46, 47].

**Abb. 3:**
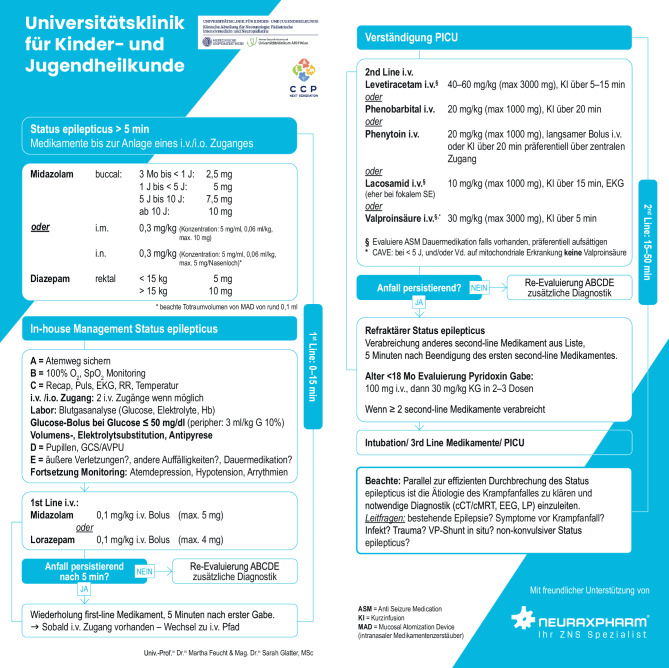
„Pocket Card PKSE“

Zeitgleich zur Klärung/zum Ausschluss behandelbarer Ursachen muss unmittelbar und ohne auf Untersuchungsergebnisse zu warten, mit der Behandlung begonnen werden. Das in Abb. 3 gezeigte strukturelle Therapie- und Diagnostikkonzept wurde entsprechend aktueller Datenlage entworfen, um unabhängig von der epileptologischen Expertise rasch strukturierte und spezifische Therapie zu ermöglichen und steht als „Pocket Card“ zur Verfügung.

Die initiale Behandlung des PKSE erfolgt (im Idealfall prähospital spätestens zum Zeitpunkt T1 und daher oft ohne Möglichkeit eines i.v.-Zuganges) wie bei Erwachsenen mit Benzodiazepinen (BDZ). Die sukzessive Verabreichung von mehr als 2 Maximaldosen wird als wirkungslos berichtet und ist mit einem erhöhten Risiko für Atemdepression assoziiert [4, 48]. Vorsicht ist bei tonischen Anfällen geboten, da BDZ in sehr seltenen Fällen tonische KSE (z. B. bei Lennox-Gastaut-Syndrom) auslösen oder aggravieren können [49–51].

Da es trotz vielfacher Warnungen vor negativen Effekten von (zu) spät initiierter oder ineffizienter Behandlung immer noch häufig zu beträchtlichen Verzögerungen kommt, wird rezent bereits bei Auftreten von prolongierten Anfällen > 2 min Dauer und bei Anfalls-Clustern (definiert als signifikante Zunahme der Anfallsfrequenz verglichen mit der für den individuellen Patienten üblichen „Baseline“), also bereits vor T1 (Entstehen eines KSE), die Gabe der Notfallmedikation (i. e. „rapid and early seizure termination“ [REST] bzw. „acute cluster treatment“ [ACT]) vorgeschlagen [8, 20, 48, 52, 53].

Für allgemeingültige Empfehlungen im (frühen) Kindesalter sind jedoch weitere prospektive kontrollierte Studiendaten notwendig. Voraussetzung ist zudem die eingehende individuelle Beratung der Angehörigen in der Epilepsiesprechstunde, um den individuellen Bedarf (inklusive die Definition von Anfalls-Clustern) festzulegen und zu niedrige BDZ-Dosen mit etwaigen negativen Konsequenzen zu vermeiden.

Eine Aufstellung der zur Verfügung stehenden antikonvulsiv wirksamen Substanzen für die Behandlung nach Versagen der Erstlinienmedikation (Zeitpunkt T2) ist ebenfalls der „Pocket Card“ zu entnehmen. Empfohlen sind Phenobarbital (PB), Phenytoin (PHT), Valproat (VPA) und Levetiracetam (LEV), wobei die Anwendung von PB aktuell primär auf Neonaten beschränkt ist sowie auf Kinder, die PHT bereits als Dauerprophylaxe einnehmen und/oder bei denen PHT kontraindiziert ist (z. B. Kinder mit Dravet-Syndrom). Aktuell wird LEV wegen des günstigeren Nebenwirkungsprofils bevorzugt und VPA – insbesondere bei Erstmanifestation des PKSE und unbekannter Ätiologie (i. e. Möglichkeit einer Mitochondriopathie) – bei Kindern < 4 Jahren möglichst vermieden [4, 15, 19, 22].

Für (S)RSE existieren derzeit keine allgemein akzeptierten Empfehlungen (weder über die Abfolge der Medikation, noch über Ziel und optimale Dauer der Komatherapie). Generell erfolgt die Behandlung mittels Midazolam- und/oder Barbiturat- sowie Ketamin- und/oder Propofol-Infusion unter kontinuierlichem EEG-Monitoring bzw. entsprechend spezifischer Ätiologie [8, 17, 19].

Positives Ergebnis ist in jedem Fall ausschließlich die zeitnahe Beendigung des PKSE (klinisch und im EEG)! Häufige Fehler in diesem Zusammenhang sind trotz ausreichender Evidenz, dass die Risiken fortbestehender Anfallsaktivität höher zu bewerten sind als Nebenwirkungen adäquat hoch dosierter Medikation – zu zögerliche Eskalation und/oder Unterdosierung der verwendeten Substanzen sowie die Nichtbeachtung von dem PKSE nachfolgenden non-konvulsiven Anfällen (Stellenwert des EEGs!) [19, 37].

**Abb. 4:**
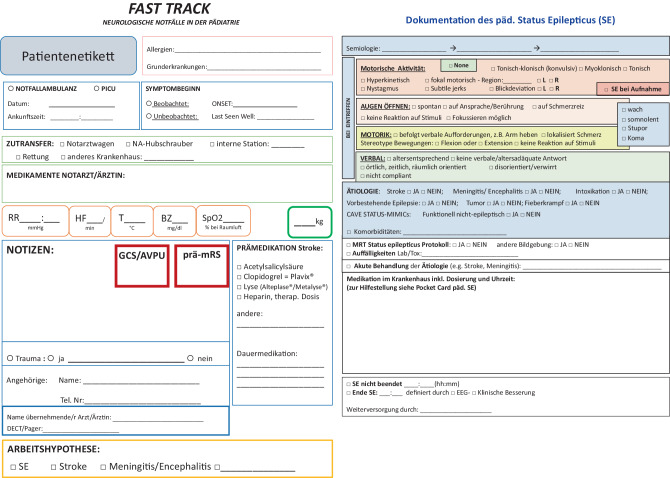
Auszug aus dem Dokumentationsformular PKSE. Mit freundlicher Genehmigung und adaptiert aus dem Dokumentationsbogen des Universitätsklinikum für Neurologie Salzburg. (*PICU* Pediatric Intensive Care Unit, *GCS* Glasgow Coma Scale, *AVPU* Alert (wach) Verbal (Reaktion auf Ansprache) Pain (Reaktion auf Schmerzreiz) Unresponsive (keine Reaktion), *prä-mRS* = Modified Rankin Scale vor Ereignis)

Ein weiterer potenziell limitierender Faktor ist bei notwendiger Transferierung von einem Spital in ein anderes (z. B. höher spezialisiertes Zentrum), aber auch bei Verlegung von der Ambulanz über die IMC an die PICU im selben Spital die fehlende Dokumentation der bislang gesetzten Maßnahmen inklusive verabreichter Medikation. Dadurch können Verzögerungen in der (Weiter‑)Behandlung entstehen. Das hier vorgestellte und abrufbare Dokumentationsformular soll dazu dienen, den Verlauf des PKSE möglichst lückenlos zu beschreiben (Abb. 4 – QR-Code).

## Prognose

Etwa 12–40 % aller PKSE im Kindesalter sprechen derzeit nicht auf First- und Second-line-Medikation an (RSE), 10–15 % dauern ab Hospitalisierung ≥ 24 h an und erfüllen somit die Kriterien eines SRSE.

Die Mortalität des PKSE beträgt 3–11 %, wobei symptomatische Ätiologien, junges Alter (i. e. < 1 Jahr), die verlängerte Zeitspanne bis Therapiebeginn und Komplikationen des PKSE bekannte Ursachen sind [5, 12, 35].

Unmittelbare Komplikationen des PKSE umfassen Tachykardie, Hypertension, respiratorisches Versagen, metabolische und/oder respiratorische Azidose, Elektrolytentgleisungen, Rhabdomyolyse, Nierenversagen sowie erhöhten Hirndruck und Hirnödem. Studien, die die Langzeitmortalität des PKSE untersuchten, kamen zu unterschiedlichen Resultaten (0–40 %). Auch hier sind Alter < 1 Jahr und symptomatische Ätiologien die meistgenannten Risikofaktoren [5, 17, 35].

Langzeitfolgen des PKSE beinhalten neurologische Ausfälle (e. g. Lähmungen, extrapyramidale und/oder zerebelläre Syndrome), kognitive Beeinträchtigungen, psychische Störungen und chronische Epilepsien. Die am meisten betroffenen Hirnstrukturen sind Hippocampus, Amygdala, dorsomedialer Thalamus und Kleinhirn [16, 29, 30, 34, 35, 40].

In der Literatur angeführte Messinstrumente zur Einschätzung der Prognose von (S)RSE sind der modified Status Epilepticus Severity Score (mSTESS) und der Status Epilepticus Pediatric Severity Score (STEPSS), wobei an der MUW mehr Erfahrung in der Verwendung des mSTESS besteht [35, 54].

## Fazit

- PKSE gehören zu den häufigsten Notfällen der Pädiatrie.
- PKSE sind „zeitsensitiv“. Rasche Diagnose und frühe spezifische Therapie sind essenziell, um Mortalität und Morbidität von PKSE hintanzuhalten.
- Obwohl die Klärung der Ätiologie notwendig ist und daher parallel ablaufen muss, ist einer der größten Fehler, z. B. durch Warten auf Befunde Zeit bis zum Beginn der Behandlung zu verlieren.
- Zweiter Fehler ist die Wahl ungeeigneter Substanzen und/oder zu niedrige Dosierungen.
- Das EEG kann sowohl bei Diagnose und Klassifikation des drohenden/initialen als auch bei Überwachung der Therapie des etablierten PKSE unterstützen. Mittelfristiges Ziel sollte daher der zeitnahe Zugang zu EEG-Langzeitableitungen sein.
- Die Zeitspanne, ab wann therapeutisch interveniert werden soll, wurde rezent sukzessive verkürzt. Aktuell besteht ein zunehmender Trend, Akuttherapie mit schnell wirksamen Substanzen noch früher (bereits bei prologierten Krampfanfällen ab ≥ 2 min Dauer und Anfalls-Clustern) zu verabreichen, um das Entstehen von etablierten PKSE im Vorfeld zu verhindern.
- Bei bekannter Neigung zu PKSE und/oder Anfalls-Clustern sollte das Umfeld der jungen PatientInnen bezüglich einer frühen BDZ-Intervention in den ersten Minuten geschult werden.
